# Crystal structure of poly[(aceto­nitrile-κ*N*)(μ_3_-7-{[bis­(pyridin-2-ylmeth­yl)amino]­meth­yl}-8-hy­droxy­quinoline-5-sulfonato-κ^4^ *N*,*O*:*O*′:*O*′′)sodium]

**DOI:** 10.1107/S2056989023005959

**Published:** 2023-07-14

**Authors:** Koji Kubono, Ryoichi Tanaka, Yukiyasu Kashiwagi, Keita Tani, Kunihiko Yokoi

**Affiliations:** a Osaka Kyoiku University, 4-698-1 Asahigaoka, Kashiwara, Osaka 582-8582, Japan; b Osaka Research Institute of Industrial Science and Technology, 1-6-50 Morinomiya, Joto-ku, Osaka 536-8553, Japan; Universität Greifswald, Germany

**Keywords:** crystal structure, coordination polymer, sodium complex, 8-hy­droxy­quinoline sulfonato, C—H⋯O inter­actions

## Abstract

In the title compound, [Na(C_22_H_19_N_4_O_4_S)(CH_3_CN)]_*n*_, the Na^I^ atom adopts a distorted square-pyramidal coordination geometry, formed by one N and one O atom of the qunolinol moiety in the ligand, two O atoms of sulfonate moieties of two adjacent ligands and the N atom of the coordinated aceto­nitrile solvent. The Na^I^ atom is located well above the mean basal plane of the square-based pyramid. The apical position is occupied by a sulfonate O atom of a neighboring ligand. Three N atoms of the bis­(pyridin-2-ylmeth­yl)amine moiety in the ligand are not coordinated by the sodium atom. The mol­ecule forms an intra­molecular bifurcated O—H⋯[N(tertiary amine),N(pyridine)] hydrogen bond, generating *S*(6) and *S*(5) rings. In the crystal, four mol­ecules are linked by four Na—O(sulfonato) bridged coordination bonds, forming a supra­molecular centrosymmetric tetra­mer unit comprising an eight-membered ring, and generating a two-dimensional network sheet. The mol­ecules of different sheets form inter­molecular C—H⋯O hydrogen bonds, and thereby a three-dimensional network structure.

## Abstract 2

In the title compound, the Na^I^ atom has a distorted square-pyramidal coordination environment. The mol­ecular structure exhibits an intra­molecular bifurcated O—H⋯[N(tertiary amine), N(pyrid­yl)] hydrogen bond. In the crystal, the mol­ecules are linked by the bridging Na—*O*(sulfonato) coordination bonds and the inter­molecular C—H⋯O hydrogen bonds, forming a three-dimensional network structure.

## 1. Chemical context

8-Quinolinol (Hq) is a well-known chelating ligand and analytical reagent (Wiberley *et al.*, 1949 ▸). Metal complexes with Hq derivatives have been investigated as pharmaceutical treatments (Mo *et al.*, 2021 ▸), magnetic materials (Ma *et al.*, 2021 ▸) and organic light-emitting diodes (Huo *et al.* 2015 ▸; Back *et al.*, 2016 ▸). As part of our research into the development of fluorescent chelate reagents for the determination of metal ions and anions, we synthesized the penta­dentate ligand, 7-{[bis-(pyridin-2-ylmeth­yl)amino]­meth­yl}-5-chloro­quinolin-8-ol (HClqdpa) containing Hq and bis(pyridin-2-ylmeth­yl)amine ­[di-(2-picol­yl)amine] (dpa) moieties (RUTSIK; Kubono *et al.*, 2015 ▸). This ligand has only rather poor water solubility. To improve the solubility, we synthesized a new and now water-soluble fluorescent chelate reagent, based on Hq containing sulfonato-sodium and dpa moieties. Herein we report the respective synthesis and the crystal structure of its aceto­nitrile solvate complex.

## 2. Structural commentary

**Figure 1:**
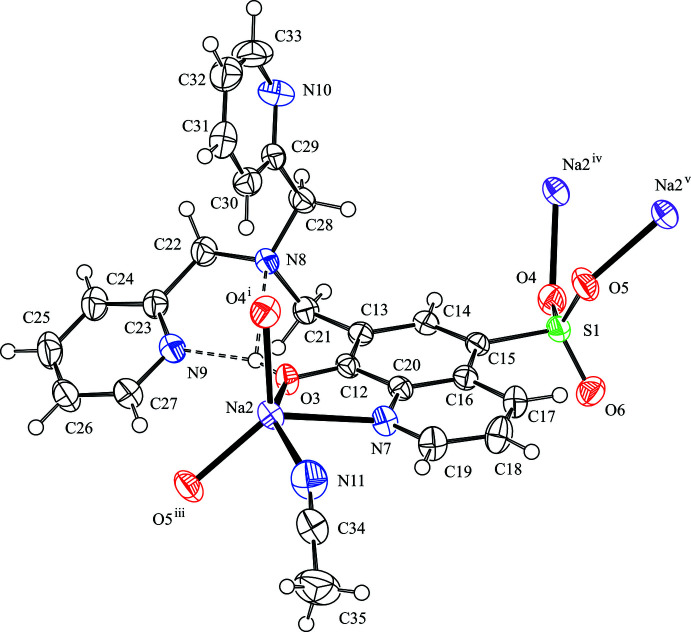
The mol­ecular structure of the title compound with atom labeling. Displacement ellipsoids are drawn at the 50% probability level. H atoms are represented by spheres of arbitrary radius. The intra­molecular O—H⋯N hydrogen bonds are shown as double-dashed lines. [Symmetry codes: (i) 2 − *x*, *y* − 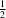 , 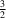 − *z*; (iii) *x*, 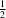 − *y*, *z* − 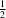 ; (iv) 2 − *x*, *y* + 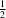 , 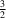 − *z*; (v) *x*, 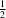 − *y*, *z* + 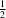 .]

The mol­ecular structure of the title compound is shown in Fig. 1 ▸. The Na^I^ atom (Na2) of the asymmetric unit adopts a distorted square-pyramidal geometry and coordinates N and O atoms of the quinolinol moiety in the ligand, two O atoms of the sulfonate moieties of two neighboring ligands and the N atom of aceto­nitrile solvent. The phenolic hydrogen atom H3 of the quinolinol moiety is bound to the O3 atom. The proton, therefore, does not dissociate. Three N atoms of the dpa moiety in the ligand are not coordinated by the Na^I^ atom. 
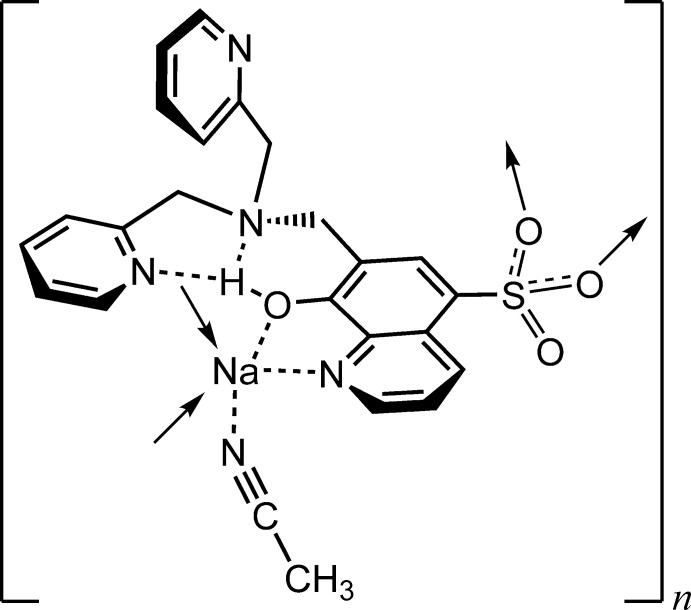


The five-coordinate geometry index, *τ* = (*β* − *α*)/60, derived from the two largest angles (*α*, *β*) in a structure has ideal values of 0 for square-pyramidal and of 1 for trigonal–bipyramidal geometry (Addison *et al.*, 1984 ▸). In the title compound it is equal to 0.310. The Na^I^ atom is located 0.7311 (8) Å above the mean basal plane [O3/N7/N11/O5^iii^; symmetry code: (iii) *x*, 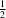 − *y*, *z* − 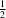 ] of the square-based pyramid. The apical position is occupied by the O4^i^ atom of the sulfonate moiety in a neighboring ligand with the Na2—O4^i^ bond being 2.2602 (16) Å long [symmetry code: (i) 2 − *x*, *y* − 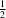 , 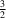 − *z*]. The Na2—O3(quinolinol) bond distance is 2.4248 (15) Å, longer than the equatorial Na—O(sulfonato) bond [Na2—O5^iii^; 2.2500 (16) Å]. The Na2—N7(quinolinol) distance is 2.467 (2) Å, shorter than the Na2—N11(aceto­nitrile) bond [2.487 (2) Å]. The chelate angle O3—Na2—N7 is 65.83 (5)°, the smallest of all the coordination angels. It agrees well with that of a related compound, (8-hy­droxy­quinoline-5-sulfonato-*N* ^1^,*O* ^8^)sodium(I) [UGUNOZ; Baskar Raj *et al.*, 2002 ▸; O—Na—N; 64.86 (4)°]. The *τ*-parameter of this related compound is 0.505, and indicative of a significantly distorted trigonal–bipyramidal geometry with bond distances of Na—O(quinolinol) and Na—N(quinolinol) of 2.4892 (14) and 2.4418 (15) Å, respectively.

The title mol­ecule forms in its crystal structure an intra­molecular bifurcated O3—H3⋯(N8, N9) hydrogen bond (Table 1 ▸), resulting in *S*(6) and *S*(5) rings, which stabilize the conformation of the mol­ecule. The N10 atom in the pyridine ring is not engaged in a coordination bond, hydrogen bond or any other inter- or intra­molecular inter­action. The dihedral angle between two pyridine rings in the title compound is 88.37 (11)°. In a related compound, 7-{[bis­(pyridin-2-ylmeth­yl)amino]­meth­yl}-5-chloro­quinolin-8-ol, HClqdpa (RUTSIK; Kubono *et al.*, 2015 ▸), the dihedral angle between two pyridine rings is 80.97 (12)°.

Even though in HClqdpa the dpa moiety is metal-free, and only one pyridine N atom forms an intra­molecular hydrogen bond with the OH group, these angles are relatively similar. The quinoline ring of the title compound is slightly bent, with r.m.s. deviations of 0.020 (2) Å. The S—O bond distances are in the range 1.4469 (14)–1.4585 (15) Å, with O—S—O angles ranging from 112.87 (9) to 113.25 (9)°. The bond lengths and angles largely agree with those values in the related compound [UGUNOZ; Baskar Raj *et al.*, 2002 ▸; S—O; 1.4482 (12)–1.4731 (12) Å, O—S—O; 110.92 (7)–114.35 (7)°]. The O6 atom is not coordinated by the Na^I^ atom, and the bond distance S1—O6 is shorter than the other two.

## 3. Supra­molecular features

**Figure 2:**
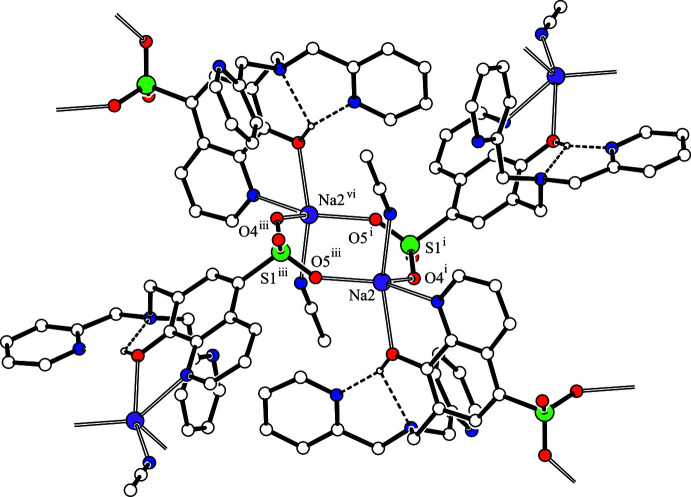
Supra­molecular centrosymmetric tetra­meric component of the crystal packing motif in the title compound formed by bridging coordination bonds. The intra­molecular hydrogen bonds are shown as dashed lines. H atoms not involved in the inter­actions are omitted for clarity. [Symmetry code: (i) 2 − *x*, *y* − 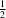 , 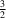 − *z*; (iii) *x*, 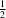 − *y*, *z* − 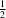 ; (vi) 2 − *x*, −*y*, 1 − *z*.]

**Figure 3:**
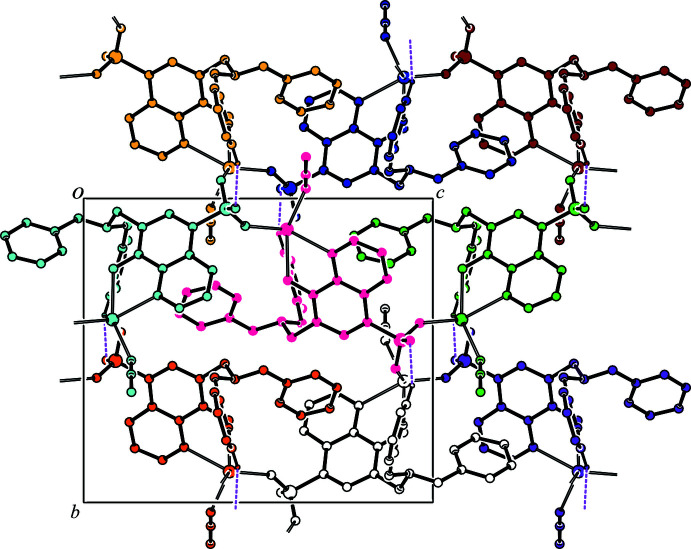
A projection along the *a* axis of the crystal packing of the title compound. The C—H⋯O hydrogen bonds are shown as dashed magenta lines. H atoms not involved in the inter­actions are omitted for clarity.

**Figure 4:**
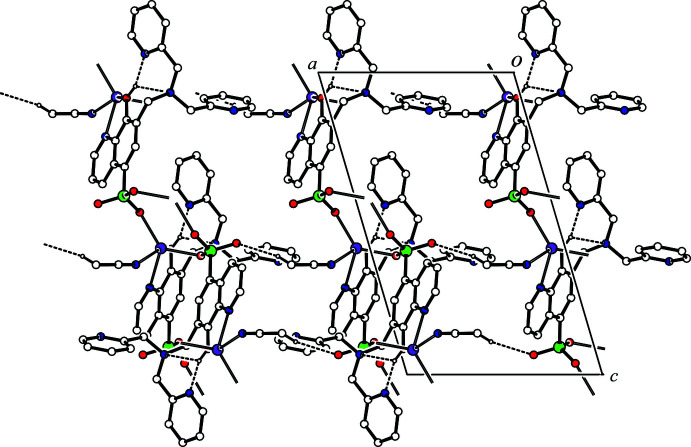
A projection along the *b* axis of the crystal packing of the title compound. The O—H⋯N and C—H⋯O hydrogen bonds are shown as dashed lines. H atoms not involved in the inter­actions are omitted for clarity.

In the crystal, four mol­ecules of the title compound are linked by four bridging Na—O coordination bonds, forming a supra­molecular centrosymmetric structure based on a central eight-membered ring (Na2/O4^i^/S1^i^/O5^i^/Na2^vi^/O4^iii^/S1^iii^/O5^iii^) [symmetry code: (vi) 2 − *x*, −*y*, 1 − *z*]. The tetra­meric building block is shown in Fig. 2 ▸. A two-dimensional coordination polymer is formed by bridging coordination bonds between the Na^I^ atom and two sulfonato O atoms of two adjacent ligands (Na2—O4^i^ and Na2—O5^iii^) in the *bc* plane (Fig. 3 ▸). An inter­molecular C—H⋯O hydrogen bond (C31—H31⋯O6^i^, Table 1 ▸) is observed, forming a *C*(12) chain motif along the *b-*axis direction. In the crystal structure, mol­ecules are further linked by an inter­molecular C—H⋯O hydrogen bond [C35—H35*A*⋯O6^ii^; symmetry code: (ii) 3 − *x*, *y* − 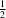 , 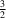 − *z*] (Table 1 ▸), forming a *C*(8) chain motif running along the *a-*axis direction (Fig. 4 ▸). The mol­ecules are linked through the bridging Na2—O4^i^ and Na2—O5^iii^ coordination bonds and the inter­molecular C35—H35*A*⋯O6^ii^ hydrogen bonds, forming a three-dimensional network structure.

## 4. Database survey

A search of the Cambridge Structural Database (CSD, Version 5.44; April 2023; Groom *et al.*, 2016 ▸) using *ConQuest* (Bruno *et al.*, 2002 ▸) for the quinolin-8-ol-5-sulfonato fragment gave 78 hits. Of these, only two structures are Na^I^ complexes with the quinolin-8-ol-5-sulfonato ligand, *viz*. (8-hy­droxy­quinoline-5-sulfonato-*N* ^1^,*O* ^8^)sodium(I) (UGUNOZ; Baskar Raj *et al.*, 2002 ▸) and its trihydrate (BOXKOO; Viossat *et al.*, 1982 ▸). Both the anhydrate and trihydrate of (quinolin-8-ol-5-sulfonato)­sodium form centrosymmetric dimeric structures in their crystals. Centrosymmetric dimer structures are observed in the crystals of various metal complexes with quinolin-8-ol-5-sulfonate and its derivatives. In the crystal of the anhydrous sodium complex, four Na—O(sulfonato) bridged coordination bonds construct a supra­molecular centrosymmetric eight-membered ring, similar to the title complex. A search for the fragment of 7-methyl-quinolin-8-ol-5-sulfonato gave two hits, which are 8-hy­droxy-7-[(morpholin-4-ium-4-yl)meth­yl]quin­oline-5-sulfonate aceto­nitrile solvate (UPAYIW; Kumar *et al.*, 2021 ▸) and 8-hy­droxy-7-[(piperidin-1-ium-1-yl)meth­yl]quin­oline-5-sulfonate monohydrate (UPAYOC; Kumar *et al.*, 2021 ▸). These compounds are metal-free ligands, and the crystal structures of their sodium salts or complexes are not reported. A search for a compound fragment in which the substituent is moved to the pyridyl ring, 2-methyl-quinolin-8-ol-5-sulfonato, gave two hits, namely aqua-{2,2′-[(1,4,10,13-tetra­oxa-7,16-di­aza­cyclo-octa­decane-7,16-di­yl)-bis­(methyl­ene)]bis­[8-(hy­droxy)quinoline-5-sulfonato]}-barium octa­hydrate (BINXEE; Thiele *et al.*, 2018 ▸), and 2-methyl-8-hy­droxy­quinoline-5-sulfonic acid monohydrate (MHQUSO; Merritt Jr, *et al.*, 1970 ▸).

## 5. Synthesis and crystallization

A suspension of paraformaldehyde (0.41 g, 14 mmol) and bis­(2-pyridyl­meth­yl)amine (1.99 g, 10 mmol) in 100 mL of MeOH was stirred for 18 h at room temperature. The solvent was removed *in vacuo*. To the product was added 90 mL of methanol, 8-hy­droxy­quinoline-5-sulfonic acid monohydrate (1.80 g, 10 mmol) and sodium hydroxide (0.40 g, 10 mmol) in 10 mL of water, the mixture was heated for 24 h at 353 K. The solvent was removed *in vacuo* to give an oily product, which was precipitated by addition of acetone (0.72 g, 31.4%). A small amount of crude solid was recrystallized from aceto­nitrile to obtain colorless crystals of the title compound. ^1^H NMR (CD_3_OD, 400 MHz): *δ* = 2.03 (*s*, 3H, aceto­nitrile), 3.90 (*s*, 4H), 3.97 (*s*, 2H), 7.23–7.26 (*m*, 2H), 7.56–7.59 (*dd*, *J* = 8.8 Hz, *J* = 4.4 Hz, 1H), 7.63 (*d*, *J* = 8.0 Hz, 2H), 7.75–7.78 (*td*, *J* = 8.0 Hz, *J* = 1.6 Hz, 2H), 8.22 (*s*, 1H), 8.45–8.47 (*m*, 2H), 8.81–8.83 (*dd*, *J* = 4.4 Hz, *J* = 1.6 Hz, 1H), 9.10–9.15 (*dd*, *J* = 8.8 Hz, *J* = 1.6 Hz, 1H). TG: expected weight loss for aceto­nitrile: 8.21%; found: 8.23% (around 447 to 465 K).

## 6. Refinement

Crystal data, data collection and structure refinement details are summarized in Table 2 ▸. The hy­droxy H atom was located in a difference-Fourier map and freely refined. All H atoms bound to carbon were positioned geometrically and refined using a riding model, with C—H = 0.95–0.99 Å and *U*_iso_(H) = 1.2 or 1.5*U*_eq_(C).

## Supplementary Material

Crystal structure: contains datablock(s) global, I. DOI: 10.1107/S2056989023005959/yz2037sup1.cif

Structure factors: contains datablock(s) I. DOI: 10.1107/S2056989023005959/yz2037Isup2.hkl

CCDC reference: 2279961

Additional supporting information:  crystallographic information; 3D view; checkCIF report

## Figures and Tables

**Table 1:** Hydrogen-bond geometry (Å, °)

*D*—H⋯*A*	*D*—H	H⋯*A*	*D*⋯*A*	*D*—H⋯*A*
O3—H3⋯N8	0.88 (2)	2.46 (3)	3.057 (2)	125 (2)
O3—H3⋯N9	0.88 (2)	1.87 (2)	2.7120 (19)	158 (3)
C31—H31⋯O6^i^	0.95	2.53	3.397 (3)	152
C35—H35*A*⋯O6^ii^	0.98	2.55	3.502 (4)	166

Symmetry codes: (i) 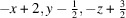 ; (ii) 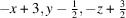 .

**Table 2:** Experimental details

Crystal data	
Chemical formula	[Na(C_22_H_19_N_4_O_4_S)(C_2_H_3_N)]
*M*_r_	499.52
Crystal system, space group	Monoclinic, *P*2_1_/*c*
Temperature (K)	173
*a*, *b*, *c* (Å)	10.4951 (4), 14.1401 (5), 16.9249 (6)
β (°)	106.378 (8)
*V* (Å^3^)	2409.77 (18)
*Z*	4
Radiation type	Mo *K*α
μ (mm^−1^)	0.19
Crystal size (mm)	0.25 × 0.20 × 0.15

Data collection	
Diffractometer	Rigaku R-AXIS RAPID
Absorption correction	Multi-scan (*ABSCOR*; Higashi, 1995 ▸)
*T*_min_, *T*_max_	0.867, 0.971
No. of measured, independent and observed [*F* ^2^ > 2.0σ(*F* ^2^)] reflections	23162, 5490, 4023
*R*_int_	0.042
(sin θ/λ)_max_ (Å^−1^)	0.648

Refinement	
*R*[*F* ^2^ > 2σ(*F* ^2^)], *wR*(*F* ^2^), *S*	0.044, 0.103, 1.01
No. of reflections	5490
No. of parameters	321
H-atom treatment	H atoms treated by a mixture of independent and constrained refinement
Δρ_max_, Δρ_min_ (e Å^−3^)	0.32, −0.30

Computer programs: *RAPID-AUTO* (Rigaku, 2006 ▸), *SIR92* (Altomare, *et al.*, 1993 ▸), *SHELXL2014/7* (Sheldrick, 2015 ▸), *PLATON* (Spek, 2020 ▸) and *CrystalStructure* (Rigaku, 2016 ▸).

## supplementary crystallographic information

### Crystal data

**Table:**

[Na(C_22_H_19_N_4_O_4_S)(C_2_H_3_N)]	*F*(000) = 1040.00
*M**_r_* = 499.52	*D*_x_ = 1.377 Mg m^−^^3^
Monoclinic, *P*2_1_/*c*	Mo *K*α radiation, λ = 0.71075 Å
*a* = 10.4951 (4) Å	Cell parameters from 16856 reflections
*b* = 14.1401 (5) Å	θ = 2.1–27.4°
*c* = 16.9249 (6) Å	µ = 0.19 mm^−^^1^
β = 106.378 (8)°	*T* = 173 K
*V* = 2409.77 (18) Å^3^	Block, colorless
*Z* = 4	0.25 × 0.20 × 0.15 mm

### Data collection

**Table:**

Rigaku R-AXIS RAPID diffractometer	4023 reflections with *F*^2^ > 2.0σ(*F*^2^)
Detector resolution: 10.000 pixels mm^-1^	*R*_int_ = 0.042
ω scans	θ_max_ = 27.4°, θ_min_ = 2.5°
Absorption correction: multi-scan (ABSCOR; Higashi, 1995)	*h* = −13→13
*T*_min_ = 0.867, *T*_max_ = 0.971	*k* = −18→18
23162 measured reflections	*l* = −20→21
5490 independent reflections	

### Refinement

**Table:**

Refinement on *F*^2^	Secondary atom site location: difference Fourier map
*R*[*F*^2^ > 2σ(*F*^2^)] = 0.044	Hydrogen site location: inferred from neighbouring sites
*wR*(*F*^2^) = 0.103	H atoms treated by a mixture of independent and constrained refinement
*S* = 1.01	*w* = 1/[σ^2^(*F*_o_^2^) + (0.0403*P*)^2^ + 1.2511*P*] where *P* = (*F*_o_^2^ + 2*F*_c_^2^)/3
5490 reflections	(Δ/σ)_max_ = 0.001
321 parameters	Δρ_max_ = 0.32 e Å^−^^3^
0 restraints	Δρ_min_ = −0.30 e Å^−^^3^
Primary atom site location: structure-invariant direct methods	

### Special details

**Table:**

Geometry. All esds (except the esd in the dihedral angle between two l.s. planes) are estimated using the full covariance matrix. The cell esds are taken into account individually in the estimation of esds in distances, angles and torsion angles; correlations between esds in cell parameters are only used when they are defined by crystal symmetry. An approximate (isotropic) treatment of cell esds is used for estimating esds involving l.s. planes.
Refinement. Refinement was performed using all reflections. The weighted R-factor (wR) and goodness of fit (S) are based on F^2^. R-factor (gt) are based on F. The threshold expression of F^2^ > 2.0 sigma(F^2^) is used only for calculating R-factor (gt).

### Fractional atomic coordinates and isotropic or equivalent isotropic displacement parameters (Å^2^)

**Table:**

	*x*	*y*	*z*	*U*_iso_*/*U*_eq_	
S1	1.18114 (5)	0.46635 (3)	0.90911 (3)	0.03024 (12)	
Na2	1.07043 (8)	0.10172 (5)	0.58208 (5)	0.03309 (19)	
O3	1.02142 (14)	0.26904 (9)	0.58507 (8)	0.0319 (3)	
O4	1.12279 (14)	0.55994 (10)	0.89339 (9)	0.0394 (3)	
O5	1.12432 (16)	0.40982 (11)	0.96284 (9)	0.0444 (4)	
O6	1.32483 (13)	0.46752 (10)	0.93467 (9)	0.0395 (3)	
N7	1.18687 (17)	0.18141 (11)	0.71220 (10)	0.0315 (4)	
N8	0.78852 (16)	0.40512 (11)	0.56663 (9)	0.0291 (3)	
N9	0.85965 (17)	0.32740 (12)	0.43831 (10)	0.0363 (4)	
N10	0.49005 (19)	0.36242 (14)	0.62557 (13)	0.0472 (5)	
N11	1.2229 (2)	−0.03275 (15)	0.63619 (14)	0.0582 (6)	
C12	1.05317 (18)	0.31647 (13)	0.65748 (11)	0.0261 (4)	
C13	1.00738 (18)	0.40634 (12)	0.66745 (11)	0.0269 (4)	
C14	1.04797 (18)	0.44926 (13)	0.74581 (11)	0.0272 (4)	
H14	1.014363	0.510196	0.752534	0.033*	
C15	1.13363 (18)	0.40683 (13)	0.81249 (11)	0.0267 (4)	
C16	1.18340 (18)	0.31472 (13)	0.80365 (11)	0.0275 (4)	
C17	1.2732 (2)	0.26440 (14)	0.86880 (12)	0.0349 (5)	
H17	1.301928	0.291025	0.922443	0.042*	
C18	1.3174 (2)	0.17810 (15)	0.85359 (13)	0.0421 (5)	
H18	1.378379	0.144070	0.896296	0.051*	
C19	1.2722 (2)	0.13945 (14)	0.77409 (13)	0.0388 (5)	
H19	1.305452	0.079314	0.764561	0.047*	
C20	1.14255 (18)	0.26954 (12)	0.72610 (11)	0.0260 (4)	
C21	0.91430 (19)	0.45661 (13)	0.59585 (12)	0.0303 (4)	
H21A	0.896845	0.521199	0.612841	0.036*	
H21B	0.956231	0.462263	0.550483	0.036*	
C22	0.7130 (2)	0.43493 (15)	0.48490 (12)	0.0364 (5)	
H22A	0.716550	0.504736	0.481568	0.044*	
H22B	0.618938	0.416526	0.475797	0.044*	
C23	0.7636 (2)	0.39248 (14)	0.41728 (12)	0.0324 (4)	
C24	0.7072 (2)	0.42029 (16)	0.33616 (13)	0.0409 (5)	
H24	0.640408	0.467689	0.323134	0.049*	
C25	0.7503 (2)	0.37761 (18)	0.27478 (13)	0.0487 (6)	
H25	0.713422	0.395451	0.218858	0.058*	
C26	0.8473 (2)	0.30886 (17)	0.29556 (14)	0.0475 (6)	
H26	0.877040	0.277717	0.254261	0.057*	
C27	0.9004 (2)	0.28629 (17)	0.37764 (14)	0.0437 (5)	
H27	0.968416	0.239856	0.392049	0.052*	
C28	0.7092 (2)	0.41116 (14)	0.62489 (12)	0.0329 (4)	
H28A	0.666499	0.474147	0.619956	0.040*	
H28B	0.768646	0.405127	0.681603	0.040*	
C29	0.60371 (19)	0.33608 (14)	0.61095 (12)	0.0313 (4)	
C30	0.6250 (2)	0.24534 (14)	0.58610 (13)	0.0375 (5)	
H30	0.706913	0.229419	0.575984	0.045*	
C31	0.5256 (2)	0.17819 (16)	0.57619 (13)	0.0448 (5)	
H31	0.537800	0.115726	0.558990	0.054*	
C32	0.4090 (2)	0.20405 (19)	0.59181 (15)	0.0523 (6)	
H32	0.338984	0.159710	0.586228	0.063*	
C33	0.3958 (2)	0.2952 (2)	0.61563 (18)	0.0590 (7)	
H33	0.314422	0.312303	0.625879	0.071*	
C34	1.3112 (3)	−0.07824 (16)	0.63605 (14)	0.0474 (6)	
C35	1.4259 (3)	−0.1369 (2)	0.6367 (2)	0.0806 (10)	
H35A	1.481852	−0.103827	0.608024	0.121*	
H35B	1.395569	−0.196938	0.608878	0.121*	
H35C	1.477330	−0.149253	0.693795	0.121*	
H3	0.958 (2)	0.2949 (17)	0.5457 (16)	0.051 (7)*	

### Atomic displacement parameters (Å^2^)

**Table:**

	*U*^11^	*U*^22^	*U*^33^	*U*^12^	*U*^13^	*U*^23^
S1	0.0311 (2)	0.0346 (3)	0.0258 (2)	−0.0037 (2)	0.00925 (19)	−0.00541 (19)
Na2	0.0414 (4)	0.0325 (4)	0.0282 (4)	−0.0002 (3)	0.0143 (3)	−0.0019 (3)
O3	0.0414 (8)	0.0307 (7)	0.0218 (7)	0.0043 (6)	0.0058 (6)	0.0001 (5)
O4	0.0424 (8)	0.0370 (8)	0.0375 (8)	0.0038 (6)	0.0093 (7)	−0.0093 (6)
O5	0.0543 (10)	0.0549 (9)	0.0285 (8)	−0.0145 (8)	0.0190 (7)	−0.0051 (6)
O6	0.0324 (7)	0.0416 (8)	0.0412 (8)	−0.0034 (6)	0.0048 (6)	−0.0066 (7)
N7	0.0405 (9)	0.0257 (8)	0.0282 (9)	0.0011 (7)	0.0095 (7)	0.0010 (6)
N8	0.0304 (8)	0.0344 (8)	0.0232 (8)	0.0014 (7)	0.0088 (7)	0.0002 (6)
N9	0.0389 (10)	0.0418 (10)	0.0293 (9)	0.0014 (8)	0.0112 (8)	−0.0003 (7)
N10	0.0402 (10)	0.0538 (12)	0.0549 (12)	0.0031 (9)	0.0255 (9)	0.0010 (9)
N11	0.0693 (15)	0.0459 (12)	0.0577 (14)	0.0152 (11)	0.0152 (11)	−0.0014 (10)
C12	0.0290 (9)	0.0279 (9)	0.0238 (9)	−0.0044 (7)	0.0112 (8)	−0.0003 (7)
C13	0.0265 (9)	0.0287 (9)	0.0266 (9)	−0.0023 (7)	0.0093 (8)	0.0021 (7)
C14	0.0285 (9)	0.0265 (9)	0.0285 (9)	−0.0006 (7)	0.0112 (8)	−0.0006 (7)
C15	0.0264 (9)	0.0303 (9)	0.0255 (9)	−0.0039 (7)	0.0106 (8)	−0.0035 (7)
C16	0.0281 (9)	0.0298 (9)	0.0258 (9)	−0.0046 (8)	0.0096 (8)	0.0020 (7)
C17	0.0411 (12)	0.0346 (11)	0.0258 (10)	−0.0037 (9)	0.0044 (9)	0.0001 (8)
C18	0.0497 (13)	0.0323 (11)	0.0354 (12)	0.0048 (10)	−0.0026 (10)	0.0054 (9)
C19	0.0490 (13)	0.0281 (10)	0.0361 (11)	0.0064 (9)	0.0066 (10)	0.0025 (8)
C20	0.0294 (9)	0.0261 (9)	0.0249 (9)	−0.0023 (7)	0.0115 (8)	0.0019 (7)
C21	0.0337 (10)	0.0292 (10)	0.0281 (10)	−0.0002 (8)	0.0090 (8)	0.0027 (8)
C22	0.0367 (11)	0.0412 (11)	0.0295 (11)	0.0061 (9)	0.0061 (9)	0.0025 (9)
C23	0.0343 (11)	0.0340 (10)	0.0275 (10)	−0.0041 (9)	0.0065 (8)	0.0010 (8)
C24	0.0452 (12)	0.0435 (12)	0.0306 (11)	−0.0026 (10)	0.0053 (9)	0.0036 (9)
C25	0.0575 (15)	0.0606 (15)	0.0256 (11)	−0.0131 (12)	0.0079 (10)	0.0011 (10)
C26	0.0533 (14)	0.0608 (15)	0.0327 (12)	−0.0085 (12)	0.0191 (11)	−0.0100 (10)
C27	0.0448 (13)	0.0514 (13)	0.0371 (12)	−0.0003 (11)	0.0153 (10)	−0.0065 (10)
C28	0.0358 (11)	0.0349 (11)	0.0305 (10)	0.0031 (8)	0.0133 (9)	−0.0036 (8)
C29	0.0312 (10)	0.0397 (11)	0.0233 (9)	0.0025 (8)	0.0081 (8)	0.0029 (8)
C30	0.0357 (11)	0.0387 (11)	0.0372 (12)	0.0026 (9)	0.0087 (9)	−0.0010 (9)
C31	0.0507 (14)	0.0426 (12)	0.0350 (12)	−0.0069 (10)	0.0021 (10)	0.0012 (9)
C32	0.0462 (14)	0.0641 (16)	0.0454 (14)	−0.0189 (12)	0.0112 (11)	0.0054 (12)
C33	0.0385 (13)	0.0741 (19)	0.0722 (19)	−0.0056 (13)	0.0286 (13)	0.0041 (15)
C34	0.0618 (16)	0.0419 (13)	0.0405 (13)	0.0019 (12)	0.0179 (12)	0.0003 (10)
C35	0.074 (2)	0.087 (2)	0.092 (2)	0.0236 (18)	0.0406 (19)	−0.0027 (19)

### Geometric parameters (Å, º)

**Table:**

S1—O6	1.4469 (14)	C17—H17	0.9500
S1—O4	1.4510 (15)	C18—C19	1.405 (3)
S1—O5	1.4585 (15)	C18—H18	0.9500
S1—C15	1.7808 (18)	C19—H19	0.9500
S1—Na2^i^	3.2984 (9)	C21—H21A	0.9900
Na2—O5^ii^	2.2500 (16)	C21—H21B	0.9900
Na2—O4^iii^	2.2602 (16)	C22—C23	1.515 (3)
Na2—O3	2.4248 (15)	C22—H22A	0.9900
Na2—N7	2.4690 (18)	C22—H22B	0.9900
Na2—N11	2.487 (2)	C23—C24	1.390 (3)
Na2—Na2^iv^	3.9829 (15)	C24—C25	1.383 (3)
O3—C12	1.354 (2)	C24—H24	0.9500
O3—H3	0.88 (3)	C25—C26	1.380 (3)
N7—C19	1.312 (3)	C25—H25	0.9500
N7—C20	1.373 (2)	C26—C27	1.380 (3)
N8—C22	1.449 (2)	C26—H26	0.9500
N8—C28	1.461 (2)	C27—H27	0.9500
N8—C21	1.466 (2)	C28—C29	1.504 (3)
N9—C23	1.337 (3)	C28—H28A	0.9900
N9—C27	1.350 (3)	C28—H28B	0.9900
N10—C29	1.338 (3)	C29—C30	1.388 (3)
N10—C33	1.347 (3)	C30—C31	1.386 (3)
N11—C34	1.129 (3)	C30—H30	0.9500
C12—C13	1.386 (3)	C31—C32	1.372 (3)
C12—C20	1.433 (3)	C31—H31	0.9500
C13—C14	1.411 (3)	C32—C33	1.369 (4)
C13—C21	1.504 (3)	C32—H32	0.9500
C14—C15	1.367 (3)	C33—H33	0.9500
C14—H14	0.9500	C34—C35	1.459 (4)
C15—C16	1.427 (3)	C35—H35A	0.9800
C16—C20	1.413 (3)	C35—H35B	0.9800
C16—C17	1.423 (3)	C35—H35C	0.9800
C17—C18	1.356 (3)		
			
O6—S1—O4	113.25 (9)	C17—C18—H18	120.3
O6—S1—O5	113.25 (9)	C19—C18—H18	120.3
O4—S1—O5	112.87 (9)	N7—C19—C18	123.96 (19)
O6—S1—C15	106.16 (9)	N7—C19—H19	118.0
O4—S1—C15	105.55 (9)	C18—C19—H19	118.0
O5—S1—C15	104.82 (9)	N7—C20—C16	122.71 (17)
O6—S1—Na2^i^	139.46 (6)	N7—C20—C12	117.21 (16)
O4—S1—Na2^i^	34.63 (6)	C16—C20—C12	120.07 (16)
O5—S1—Na2^i^	79.43 (7)	N8—C21—C13	110.84 (15)
C15—S1—Na2^i^	107.18 (6)	N8—C21—H21A	109.5
O5^ii^—Na2—O4^iii^	127.68 (7)	C13—C21—H21A	109.5
O5^ii^—Na2—O3	101.42 (6)	N8—C21—H21B	109.5
O4^iii^—Na2—O3	92.56 (6)	C13—C21—H21B	109.5
O5^ii^—Na2—N7	130.30 (7)	H21A—C21—H21B	108.1
O4^iii^—Na2—N7	101.50 (6)	N8—C22—C23	113.07 (16)
O3—Na2—N7	65.83 (5)	N8—C22—H22A	109.0
O5^ii^—Na2—N11	88.66 (7)	C23—C22—H22A	109.0
O4^iii^—Na2—N11	104.46 (7)	N8—C22—H22B	109.0
O3—Na2—N11	148.91 (7)	C23—C22—H22B	109.0
N7—Na2—N11	85.14 (7)	H22A—C22—H22B	107.8
O5^ii^—Na2—S1^iii^	113.91 (5)	N9—C23—C24	122.47 (19)
O4^iii^—Na2—S1^iii^	21.39 (4)	N9—C23—C22	118.05 (17)
O3—Na2—S1^iii^	112.83 (4)	C24—C23—C22	119.46 (19)
N7—Na2—S1^iii^	115.19 (5)	C25—C24—C23	118.7 (2)
N11—Na2—S1^iii^	88.83 (6)	C25—C24—H24	120.6
O5^ii^—Na2—Na2^iv^	57.30 (5)	C23—C24—H24	120.6
O4^iii^—Na2—Na2^iv^	76.32 (5)	C26—C25—C24	119.3 (2)
O3—Na2—Na2^iv^	132.85 (5)	C26—C25—H25	120.3
N7—Na2—Na2^iv^	160.89 (5)	C24—C25—H25	120.3
N11—Na2—Na2^iv^	77.20 (6)	C25—C26—C27	118.6 (2)
S1^iii^—Na2—Na2^iv^	57.86 (2)	C25—C26—H26	120.7
C12—O3—Na2	120.18 (11)	C27—C26—H26	120.7
C12—O3—H3	114.9 (16)	N9—C27—C26	122.8 (2)
Na2—O3—H3	120.7 (16)	N9—C27—H27	118.6
S1—O4—Na2^i^	123.98 (9)	C26—C27—H27	118.6
S1—O5—Na2^v^	148.14 (10)	N8—C28—C29	112.78 (16)
C19—N7—C20	117.48 (17)	N8—C28—H28A	109.0
C19—N7—Na2	124.08 (13)	C29—C28—H28A	109.0
C20—N7—Na2	117.42 (12)	N8—C28—H28B	109.0
C22—N8—C28	111.39 (16)	C29—C28—H28B	109.0
C22—N8—C21	112.06 (15)	H28A—C28—H28B	107.8
C28—N8—C21	111.93 (15)	N10—C29—C30	122.79 (19)
C23—N9—C27	118.06 (18)	N10—C29—C28	115.47 (18)
C29—N10—C33	116.2 (2)	C30—C29—C28	121.73 (18)
C34—N11—Na2	151.4 (2)	C31—C30—C29	119.4 (2)
O3—C12—C13	124.01 (17)	C31—C30—H30	120.3
O3—C12—C20	116.23 (16)	C29—C30—H30	120.3
C13—C12—C20	119.75 (17)	C32—C31—C30	118.4 (2)
C12—C13—C14	119.07 (17)	C32—C31—H31	120.8
C12—C13—C21	120.28 (17)	C30—C31—H31	120.8
C14—C13—C21	120.64 (16)	C33—C32—C31	118.5 (2)
C15—C14—C13	122.77 (17)	C33—C32—H32	120.8
C15—C14—H14	118.6	C31—C32—H32	120.8
C13—C14—H14	118.6	N10—C33—C32	124.7 (2)
C14—C15—C16	119.26 (17)	N10—C33—H33	117.6
C14—C15—S1	119.96 (14)	C32—C33—H33	117.6
C16—C15—S1	120.78 (14)	N11—C34—C35	179.4 (3)
C20—C16—C17	117.06 (17)	C34—C35—H35A	109.5
C20—C16—C15	119.07 (17)	C34—C35—H35B	109.5
C17—C16—C15	123.87 (17)	H35A—C35—H35B	109.5
C18—C17—C16	119.36 (19)	C34—C35—H35C	109.5
C18—C17—H17	120.3	H35A—C35—H35C	109.5
C16—C17—H17	120.3	H35B—C35—H35C	109.5
C17—C18—C19	119.39 (19)		
			
O6—S1—O4—Na2^i^	−146.44 (9)	Na2—N7—C20—C12	−13.3 (2)
O5—S1—O4—Na2^i^	−16.08 (13)	C17—C16—C20—N7	−0.6 (3)
C15—S1—O4—Na2^i^	97.84 (10)	C15—C16—C20—N7	179.29 (17)
O6—S1—O5—Na2^v^	78.3 (2)	C17—C16—C20—C12	−179.41 (17)
O4—S1—O5—Na2^v^	−52.0 (2)	C15—C16—C20—C12	0.5 (3)
C15—S1—O5—Na2^v^	−166.41 (18)	O3—C12—C20—N7	−0.3 (2)
Na2^i^—S1—O5—Na2^v^	−61.26 (19)	C13—C12—C20—N7	−178.66 (16)
Na2—O3—C12—C13	−167.32 (14)	O3—C12—C20—C16	178.61 (16)
Na2—O3—C12—C20	14.4 (2)	C13—C12—C20—C16	0.2 (3)
O3—C12—C13—C14	−179.49 (17)	C22—N8—C21—C13	−163.21 (16)
C20—C12—C13—C14	−1.2 (3)	C28—N8—C21—C13	70.82 (19)
O3—C12—C13—C21	1.3 (3)	C12—C13—C21—N8	63.1 (2)
C20—C12—C13—C21	179.60 (16)	C14—C13—C21—N8	−116.12 (18)
C12—C13—C14—C15	1.6 (3)	C28—N8—C22—C23	−155.23 (16)
C21—C13—C14—C15	−179.20 (17)	C21—N8—C22—C23	78.5 (2)
C13—C14—C15—C16	−0.9 (3)	C27—N9—C23—C24	−1.4 (3)
C13—C14—C15—S1	179.41 (14)	C27—N9—C23—C22	176.90 (19)
O6—S1—C15—C14	−126.08 (15)	N8—C22—C23—N9	6.4 (3)
O4—S1—C15—C14	−5.59 (17)	N8—C22—C23—C24	−175.17 (18)
O5—S1—C15—C14	113.80 (16)	N9—C23—C24—C25	1.3 (3)
Na2^i^—S1—C15—C14	30.51 (16)	C22—C23—C24—C25	−177.0 (2)
O6—S1—C15—C16	54.26 (17)	C23—C24—C25—C26	0.1 (3)
O4—S1—C15—C16	174.74 (15)	C24—C25—C26—C27	−1.3 (3)
O5—S1—C15—C16	−65.86 (17)	C23—N9—C27—C26	0.2 (3)
Na2^i^—S1—C15—C16	−149.16 (13)	C25—C26—C27—N9	1.2 (4)
C14—C15—C16—C20	−0.1 (3)	C22—N8—C28—C29	72.0 (2)
S1—C15—C16—C20	179.53 (13)	C21—N8—C28—C29	−161.71 (16)
C14—C15—C16—C17	179.75 (18)	C33—N10—C29—C30	0.6 (3)
S1—C15—C16—C17	−0.6 (3)	C33—N10—C29—C28	−178.2 (2)
C20—C16—C17—C18	1.6 (3)	N8—C28—C29—N10	−145.01 (18)
C15—C16—C17—C18	−178.31 (19)	N8—C28—C29—C30	36.2 (3)
C16—C17—C18—C19	−0.9 (3)	N10—C29—C30—C31	−0.4 (3)
C20—N7—C19—C18	1.9 (3)	C28—C29—C30—C31	178.36 (19)
Na2—N7—C19—C18	−166.24 (17)	C29—C30—C31—C32	−0.3 (3)
C17—C18—C19—N7	−0.9 (4)	C30—C31—C32—C33	0.7 (3)
C19—N7—C20—C16	−1.1 (3)	C29—N10—C33—C32	−0.2 (4)
Na2—N7—C20—C16	167.84 (13)	C31—C32—C33—N10	−0.4 (4)
C19—N7—C20—C12	177.74 (18)		

Symmetry codes: (i) −*x*+2, *y*+1/2, −*z*+3/2; (ii) *x*, −*y*+1/2, *z*−1/2; (iii) −*x*+2, *y*−1/2, −*z*+3/2; (iv) −*x*+2, −*y*, −*z*+1; (v) *x*, −*y*+1/2, *z*+1/2.

### Hydrogen-bond geometry (Å, º)

**Table:**

*D*—H···*A*	*D*—H	H···*A*	*D*···*A*	*D*—H···*A*
O3—H3···N8	0.88 (2)	2.46 (3)	3.057 (2)	125 (2)
O3—H3···N9	0.88 (2)	1.87 (2)	2.7120 (19)	158 (3)
C31—H31···O6^iii^	0.95	2.53	3.397 (3)	152
C35—H35*A*···O6^vi^	0.98	2.55	3.502 (4)	166

Symmetry codes: (iii) −*x*+2, *y*−1/2, −*z*+3/2; (vi) −*x*+3, *y*−1/2, −*z*+3/2.
